# Autism genetic database (AGD): a comprehensive database including autism susceptibility gene-CNVs integrated with known noncoding RNAs and fragile sites

**DOI:** 10.1186/1471-2350-10-102

**Published:** 2009-09-24

**Authors:** Gregory Matuszek, Zohreh Talebizadeh

**Affiliations:** 1 K-INBRE Bioinformatics Core Facility, University of Kansas, Lawrence, KS, USA; 2 Section of Medical Genetics and Molecular Medicine, Children's Mercy Hospitals and University of Missouri-Kansas City School of Medicine, Kansas City, MO, USA

## Abstract

**Background:**

Autism is a highly heritable complex neurodevelopmental disorder, therefore identifying its genetic basis has been challenging. To date, numerous susceptibility genes and chromosomal abnormalities have been reported in association with autism, but most discoveries either fail to be replicated or account for a small effect. Thus, in most cases the underlying causative genetic mechanisms are not fully understood. In the present work, the Autism Genetic Database (AGD) was developed as a literature-driven, web-based, and easy to access database designed with the aim of creating a comprehensive repository for all the currently reported genes and genomic copy number variations (CNVs) associated with autism in order to further facilitate the assessment of these autism susceptibility genetic factors.

**Description:**

AGD is a relational database that organizes data resulting from exhaustive literature searches for reported susceptibility genes and CNVs associated with autism. Furthermore, genomic information about human fragile sites and noncoding RNAs was also downloaded and parsed from miRBase, snoRNA-LBME-db, piRNABank, and the MIT/ICBP siRNA database. A web client genome browser enables viewing of the features while a web client query tool provides access to more specific information for the features. When applicable, links to external databases including GenBank, PubMed, miRBase, snoRNA-LBME-db, piRNABank, and the MIT siRNA database are provided.

**Conclusion:**

AGD comprises a comprehensive list of susceptibility genes and copy number variations reported to-date in association with autism, as well as all known human noncoding RNA genes and fragile sites. Such a unique and inclusive autism genetic database will facilitate the evaluation of autism susceptibility factors in relation to known human noncoding RNAs and fragile sites, impacting on human diseases. As a result, this new autism database offers a valuable tool for the research community to evaluate genetic findings for this complex multifactorial disorder in an integrated format. AGD provides a genome browser and a web based query client for conveniently selecting features of interest. Access to AGD is freely available at .

## Background

Autism is an early onset neurodevelopmental disorder belonging to a group of conditions known as autism spectrum disorders (ASDs) which includes classical autism, pervasive developmental disorder-not otherwise specified (PDD-NOS), and Asperger syndrome [1]. ASDs are genetically and phenotypically heterogeneous with a variable degree of severity and symptomology. The prevalence of autism spectrum disorders has risen in recent decades to 6.7 per 1000 children in the United States [2]. Diagnosis of autism is defined by significant impairments in three developmental domains: reciprocal social behavior, communication, and repetitive stereotypic behaviors or restricted interests [1].

Several candidate genes have been linked to this highly heritable disorder, but the etiology of most cases remains unknown. Linkage analyses for autism susceptibility loci have suggested the involvement of multiple genes from different chromosomes. Despite the completion of several genome-wide linkage studies for autism, most of the loci identified have not been replicated. Furthermore, association of several candidate genes have been reported and examined in subjects with autism, mainly with no conclusive evidence. As a result, a number of autism susceptibility nucleotide changes have been reported but have not been replicated. These inconsistent results could be in part a reflection of the clinical heterogeneity and varying degrees of severity in ASD.

For example, in 2003 the first evidence of mutations in coding sequences of two X-linked neuroligin genes, *NLGN3 *and *NLGN4*, were reported in individuals with autism spectrum disorders [3]. Neuroligins are cell adhesion proteins involved in the formation of neural synapses [4]. Electrophysiological studies on mutant neuroligins carrying deletions in either the cytoplasmic tail or in the esterase-homology domain showed the critical role of the neuroligin genes in maintaining a functional balance between excitatory and inhibitory synapses in hippocampal neurons [5]. This finding resulted in the conclusion that neuroligin defects lead to selective loss of inhibitory function and abnormal excitatory/inhibitory balance in neurons. Such a defect is believed to play a role in autism [5,6].

Despite strong supportive evidence for the role of these neuroligin genes in synaptic function, only a few causal mutations in the *NLGN3 *and *NLGN4 *genes have been identified in subjects with autism, suggesting that these mutations are not common and occur at a low frequency in the autistic population (less than 1%) [7-15]. Therefore, at the population level the actual proportion of known genetic variants or changes contributing to the etiology of autism remains to be determined, since most identified genetic causes may account for a small effect. A fact that is expected, given the clinical heterogeneity and varying degrees of severity in this complex disorder, which demands the evaluation of multiple factors using integrated approaches.

Furthermore, genomic DNA copy number variations (CNVs) including small deletions and duplications of chromosomes, which may affect gene function have been recently reported in association with complex disorders such as autism [16-19]. In a recent review, the association of CNVs with neuropsychiatric conditions including ASD was discussed by Cook and Scherer [20]. One conclusion of this review paper was that while it is more likely for a *de novo *than an inherited CNV to be pathogenic, the final causal effect of CNVs might be influenced by other *cis*- or *trans*-acting factors in a particular genomic environment, representing in an incomplete penetrance or a variable expressivity for a given CNV. This suggests that due to the complexity of neuropsychiatric disorders, the evaluation of biological relevance of CNVs should be considered in an integrated context [20]. For a recent review discussing advances in autism genetics see Abrahams and Geschwind [21].

Recent developments in molecular genetic technologies and knowledge have introduced new avenues to be explored, in particular for complex disorders. A good example is gene regulatory factors such as noncoding RNAs (ncRNAs) which are highly expressed in the nervous system [22]. An estimated 98% of the transcriptional output in humans and other mammals consist of ncRNAs that do not code for protein but have other functions in cells [23]. Four main groups of ncRNAs include microRNAs, snoRNAs, piRNAs, and siRNAs. A brief description of each type and their relevance to human disease is provided here.

microRNAs are small RNA molecules of approximately 22 nt that regulate gene expression by binding to the 3'-untranslated regions (3'UTR) of target mRNA(s), directing translational repression or transcript degradation [24]. It is estimated that up to 30% of human genes may be microRNA targets [25]. Small nucleolar RNAs (snoRNAs) direct the site-specific modification of nucleotides in target ribosomal RNAs (rRNAs) [26]. However, some snoRNA (known as orphan snoRNAs) lack known targets for rRNA. Two classes of snoRNA can be distinguished based on their conserved sequence motifs: H/ACA box snoRNAs and C/D box snoRNAs. The C/D box snoRNAs contain four conserved motifs called boxes C, C', D, and D', with a 10-21 nucleotide long antisense element located upstream of the D and/or D' boxes.

One of the most studied snoRNAs in humans is HBII-52, located at chromosome 15q11 [27]. In addition to HBII-52, this chromosomal region contains several other paternally expressed (imprinted) brain-specific orphan snoRNAs [27]. However, complementarity to a given mRNA sequence has been reported for only HBII-52. The antisense element of HBII-52 exhibits an 18-nt complementarity to the *5-HT2C *mRNA whereby it is subject to posttranscriptional RNA editing and an alternatively spliced exon Vb [27]. Subjects with Prader-Willi syndrome, a neurodevelopmental disorder involving a chromosome 15q11 abnormality, have different *5-HT2C *mRNA processing than healthy individuals, which may contribute to their clinical symptoms [28]. In an attempt to identify targets for other orphan snoRNAs, we have recently developed a computer program, snoTARGET [29]. According to our initial analysis using snoTARGET, there are potential target mRNAs for other orphan snoRNAs which need to be verified using molecular and functional assays. This finding further suggests the importance of exploring the role of snoRNAs in human diseases.

piRNA (Piwi-interacting RNAs) are a newly discovered class of small RNAs, 26-31 nucleotides in length, that are expressed abundantly in the spermatogenic cells [30]. The majorities of piRNAs exist as clusters and occur on one or both strands, designated as monodirectional or bidirectional clusters, respectively. The biological function of piRNAs is not fully known, but their expression pattern indicates that they play roles in spermatogenesis and germline development [30].

Small interfering RNAs (siRNAs) are about 21 nucleotides in length and derive from double stranded RNA (dsRNA), typically a result of transgenic, viral or other exogenous dsRNA sources [31]. In addition to exogenous siRNAs, there have been reports of endogenous siRNAs found in plants, flies, and mammals [31]; however, endogenous siRNAs in humans remain to be discovered. The siRNAs consist of a guide strand and a passenger strand. The guide strand binds to mRNA molecules resulting in a knockdown in the levels of mRNA, protein or both [31]. Brief analysis of the siRNA data available from the MIT siRNA database, containing experimentally validated siRNAs [32], showed that several autism candidate genes are targets of exogenous siRNAs.

Multiple classes of ncRNAs are highly represented in the nervous system, emphasizing the likelihood that nervous system development and function is heavily dependent on RNA regulatory networks, and alterations of these networks may result in many neurological diseases. It is thought that ncRNAs may provide the key to better understanding the etiology of human diseases, particularly neurological diseases [33]. For example, dysregulation of microRNAs has been reported in association with Alzheimer's disease [34-36], Parkinson's disease [37], and Tourette's syndrome [38]. More recently, a study conducted by our group [39] and a report by Abu-Elneel et al. [40] suggested that microRNAs should be evaluated in the etiology of autism. Therefore, functional features and biological significance of ncRNAs suggests that this class of gene regulatory factors should be considered in relation to complex disorders.

Fragile sites are another important genomic factor in human genetics. Fragile chromosome sites are nonrandom gaps or breaks of variable size that can appear spontaneously or after exposing the cells to chemical agents [41,42]. Based on their frequency in the general population, fragile sites can be classified into two main classes: common and rare [42]. One rare fragile site (FRAXE) is associated with a form of mental retardation and also has been reported as the most common cause of autism [43,44]. Analysis of the global distribution of fragile sites and microRNAs in relation to genomic regions involved in cancers indicated that microRNAs are frequently located at fragile sites and cancer-associated genomic regions [45]. These lines of evidence warrant the need for further analysis of fragile sites in autism using an integrated approach to gain more insight into the possible role of this form of cytogenetic marker in relation to other contributing genetic factors.

The growing list of autism susceptibility genetic factors and the need to explore the role of gene regulatory elements (e.g., ncRNAs) warrants the implementation of bioinformatics tools to facilitate a more comprehensive approach evaluating this complex neurodevelopmental disorder. In an effort to make all reported genomic features associated with ASD (i.e., susceptibility genes and CNVs) and their potential relationship with other genomic features impacting on human disease (e.g., ncRNAs [23] and fragile sites [46]) accessible to the scientific community, the Autism Genetic Database (AGD), a freely available database, was designed by our research group.

## Construction and content

**Figure 1:**
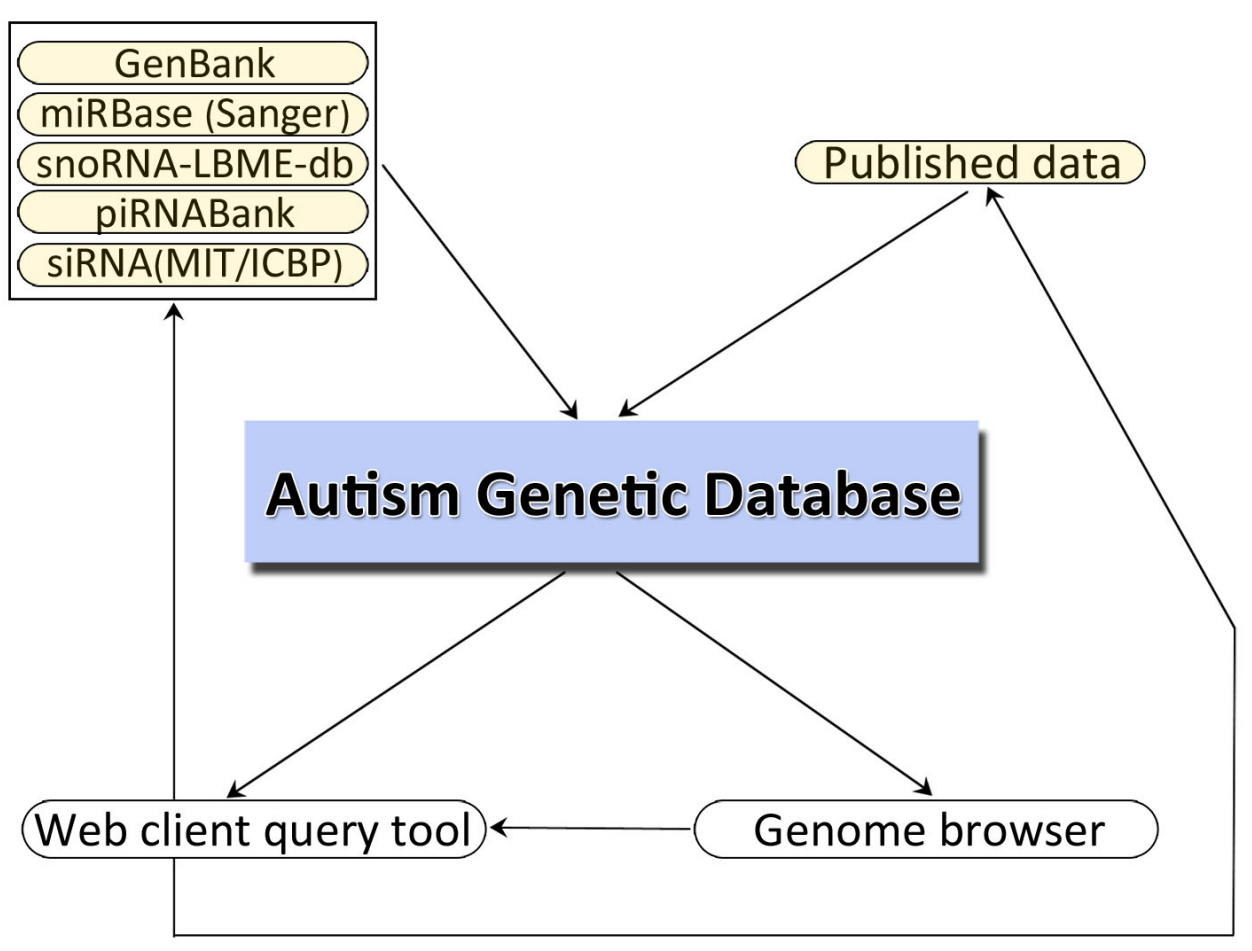
**Data flow diagram for Autism Genetic Database (AGD)**. External sources (yellow) are downloaded into AGD (blue). External data sources are also referenced from the Web client where applicable.

AGD is implemented as a mySQL (v5.0.51)  relational database with the schema for the AGD data organization available on the website. Its web interface was implemented in Perl:CGI, and HTML with the database query logic implemented in Perl:DBI. The genome browser is a modified implementation of the generic genome browser . A description of the pipeline for data collection and integration is depicted in Figure 1.

AGD stores the lists of autism susceptibility genes and CNVs generated through scientific literature searches. For the susceptibility genes, we included those with at least one suggestive report for their association with autism. Using this broad definition we identified a total of 145 genes after reviewing 1228 articles obtained from PubMed (August 2008) using the search key words "autism" and "gene". The results were carefully evaluated by the PI (Z.T.) and a three-level classification system was used for autism susceptibility genes to reflect their level of association according to the reviewed publications: 3>2>1 whereby category 3 represents the strongest autism candidate genes. This arbitrary classification of genes has been added to provide a quick reference for the importance of each gene in relation to autism while the users may decide not to consider our classification.

For CNVs, those with at least one reported association with ASD were included. *De novo *or familial status of each CNV, as well as the subject's gender was included whenever such information was available in the reference paper. Due to the lack of a standardized formal naming convention for copy number variations that provides a unique identifier for each CNV, an informal identifier has been established for each of the CNVs associated with ASD using the chromosomal band followed by a roman numeral. One such example would be the CNV 17p12_III, which is the third CNV that occurs in the chromosomal band 17p12.

The snoRNA data was obtained using the sno/scaRNA coordinate file (version hg18, NCBI Build 36.1) from sno-RNA-LBME-db [47] and the microRNA data was obtained using the miRBase sequence file (version 12.0) from miRBase [48]. piRNABank database was used as a source for all known human piRNA [49]. Access to the data stored in the piRNABank database was kindly provided by Dr. Agrawal. The siRNA data was obtained from the MIT/ICBP siRNA database (January 9, 2009 update) [32]. At this point in time, only exogenous siRNAs have been discovered for humans, in the event that endogenous human siRNAs are discovered they will be incorporated into our database. Also, because all current siRNAs arise from exogenous DNA sources, we only included those siRNA that target autism susceptibility genes. A list of human fragile sites was obtained from a review paper by Debacker and Kooy [46] and their genomic locations were retrieved from the UCSC genome browser (version hg18, NCBI Build 36.1). Applying the above criteria resulted in a total of 145 and 473 autism susceptibility genes and CNVs respectively, plus 668,688 noncoding RNAs (667,774 piRNAs, 534 microRNAs, 374, snoRNAs, and 6 siRNAs) and 120 human fragile sites, including rare fragile sites (i.e., present in a small portion of the population) and common fragile sites (i.e., present in all individuals), grouped and organized by feature type in our database. Due to the large number of piRNAs, the chromosomal views in tabular form will not display piRNAs unless explicitly selected.

## Utility and discussion

**Figure 2:**
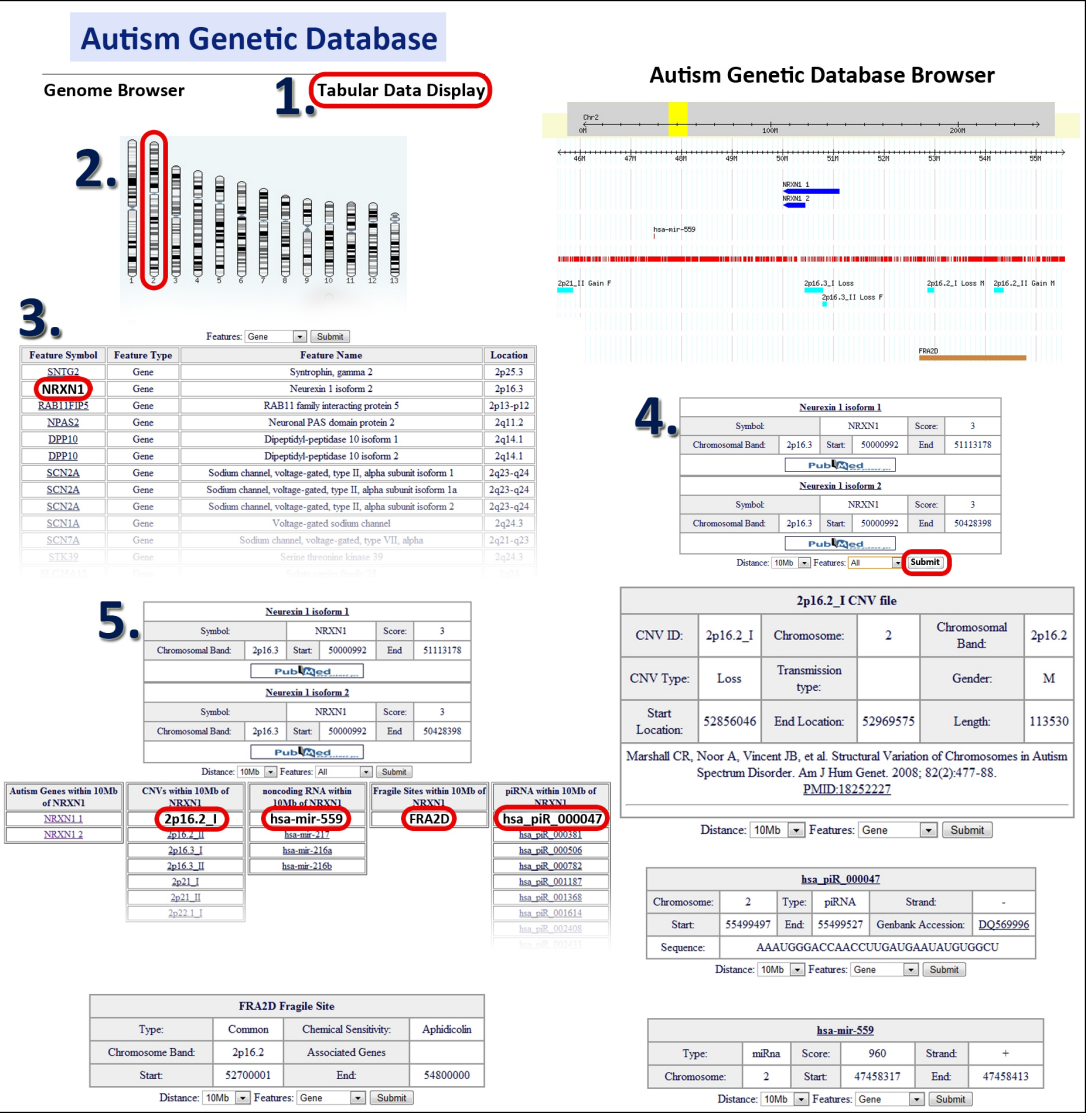
**Online display of AGD search results in both the tabular and the genome browser formats for the region surrounding the *NRXN1 *gene on chromosome 2**. The tabular display includes an example usage flow indicating the scheme to follow (shown by numbers 1 to 5) when using the database.

In AGD, data is searchable and displayed in two formats: query based tabular and genome browser. Both tools display the genes, CNVs, ncRNAs, and fragile sites in an easily accessible framework. A web-based tabular query tool enables the user to start the search from any of the above four categories of features. A combination of criteria can then be employed to display other features within a user specified distance of the selected factor's physical chromosomal location (Figure 2). Additional data relating to each feature are provided through expanded tables and through hyperlinks to the appropriate databases (e.g., PubMed, set to search with "gene name" and "autism" which provides up-to-date literature search; and miRBase for microRNAs) or literature references (e.g., for CNVs). In addition to the tabular display of data, users may also employ the customized genome browser based on the Generic Genome Browser [50] to visualize the different data tracks for the genes and CNVs related to autism as well as their surrounding ncRNAs and fragile sites.

While databases to maintain both autism susceptibility genes (AutDB [51]) and CNVs (Autism Chromosome Rearrangement Database-ACRD [52]) have been recently developed, the main function of these available resources is to serve as a catalog of the relevant subset of autism related genomic data. ACRD also offers the possibility to visualize microRNAs in relation to autism associated chromosomal abnormalities. However, in ACRD a query begins with selecting a CNV and thus it does not allow a flexible search tool as the one provided by AGD (i.e., to perform a search using a given gene, CNV, ncRNA, or fragile site). AGD was designed as a repository for all reported genetic features in association with autism, with the goal of facilitating the elucidation of possible relationships between known potential ASD elements and other genetic features. Some of these features are not yet fully evaluated, but are potentially important in the etiology of genetic disorders, such as fragile sites and noncoding RNAs.

This new autism related data resource, AGD, will be routinely updated and upgraded as new information relating to ASD becomes available. Current plans for future developments of AGD are to incorporate additional tools allowing users to correlate currently stored information with linkage analysis studies and noncoding RNA gene targets. Additionally, plans are being formalized to add SNP information into the AGD database expanding search functions and application of this autism resource for the research community.

## Conclusion

The Autism Genetic Database is a repository resource incorporating all reported autism susceptibility genes and CNVs integrated with the known human noncoding RNAs and fragile sites. The scope of the AGD database distinguishes it from other recently developed data catalogs (i.e., AutDB [51] and ACRD [52]) by incorporating non-ASD genetic factors relevant to human diseases, particularly in complex disorders. Such a comprehensive repository for genomic information relating to ASD is crucial for the advancement of computational research into the field. The web interface provided by our program enables researchers, for example, to quickly identify specific ncRNAs within or close to reported autism candidate genes or CNVs in a subset of autistic subjects with common multiple subtle genomic features. Furthermore, the availability of such an integrated and comprehensive database provides a valuable opportunity to explore and test certain autism genetic models.

## Availability and requirements

The Autism Genetic Database is freely accessible at .

## Competing interests

The authors declare that they have no competing interests.

## Authors' contributions

ZT developed and supervised the project. GM and ZT designed the web interface. GM implemented the database and built the data integration pipeline and web interface. ZT extracted and curated autism genetic information from literature. ZT and GM drafted and contributed in witting of the manuscript. All authors read and approved the final version of manuscript.

## Pre-publication history

The pre-publication history for this paper can be accessed here:


